# Comment on “Outside the Safe Operating Space of the Planetary Boundary for Novel Entities”

**DOI:** 10.1021/acs.est.2c00524

**Published:** 2022-04-15

**Authors:** Jan G Kunnas

**Affiliations:** JYU.Wisdom, School of Resource Wisdom PO Box 35, FI-40014 University of Jyväskylä Jyväskylä Finland

Persson et al. argue that we are outside the safe operating space of the planetary boundary for novel entities, since the annual production and releases of chemicals including plastics are increasing at a pace that outstrips the global capacity for assessment and monitoring.^1^ But they do not show where the boundary for novel entities is, and that it has been crossed. Instead, their argument is based upon the cautionary principle, arguing that the current growth has not been proved to be within the safe operating space. Only a fraction of the chemicals currently in use has been assessed for risk or safety. This is, however, not a planetary boundary, but a societal boundary. A measure of the ability or inability of chemical screening to keep at pace with the introduction of new chemicals and their mixtures.

We do not know, where the threshold for the safe operating space lies, but we have probably crossed it. This situation resembles our knowledge about carbon dioxide in the mid-1950s. In 1956 Canadian physicist Gilbert Plass estimated that if the carbon dioxide content of the atmosphere doubles, the surface temperature will rise by 3.6 Celsius degrees. Contrary to his predecessors, he regarded this as something to be worried about arguing that “the temperature from this cause may be so large in several centuries that it will present a serious problem to future generations”. The following year Roger Revelle and Hans E. Suess hardened the tone, stating that the present rate of combustion of fossil fuels presents “a large-scale geophysical experiment of a kind that could not have happened in the past nor be reproduced in the future.”^2^

We have now over 350 000 ongoing geophysical experiments, as this is the estimated amount of chemicals and mixtures of chemicals registered for production and use. Of these, the identities remain publicly unknown of one-third, as they are claimed as confidential or ambiguously described.^3^ Thus, it is hardly possible to condense novel entities into one or two thresholds that we should not surpass, as with other planetary boundaries.

To disperse this fog of uncertainty, we need a moratorium on taking new chemicals or their mixture into use until this backlog of chemicals available on the market without assessment of risk or safety has been cleared. The minimum requirement should be that the annual assessment should be significantly higher than the introduction of new substances. From the perspective of the planetary boundaries, this screening could have three possible outcomes. The chemical or a combination of chemicals:

(1) Is considered most likely to be safe, as full assurance is not possible due to complex interactions between a combination of chemicals and the Earth system.
(2) Is posing a threat to another planetary boundary. For example, the production cycle of plastic has climate impacts, while plastics affect biosphere integrity through physical impacts on species like entanglement or ingestion of microplastics.^4^
(3) Is posing a novel threat to the Earth system requiring the definition of a new planetary boundary.

The long-run goal should be to eliminate the Novel entities category altogether. Meanwhile, a screening at a pace faster than the introduction of new chemicals would decrease this backlog.

There are many approaches to ramping up the evaluations for chemicals with unknown effects already in use. For example, read-across approaches can be used for the safety assessment of compounds that have similar structures or result in the same major metabolites predicting their toxicity without experimental testing.^5^ We also need coordinated global efforts to avoid unnecessary duplicate testing, but this should be done in a way that does not lower safety requirements to the lowest global level. In this regard, the suggested global science-policy body similar to the IPCC on chemicals and waste would be a great step forward.^6^

Unfortunately, a normal screening of toxicity and persistence is not enough to rule out unforeseen consequences. Chlorofluorocarbons were, for example, until 1974 just a group of nontoxic, colorless, odorless, nonflammable, and noncorrosive chemicals considered mostly harmless. This changed as Mario Molina and Frank Rowland showed that the intense ultraviolet radiation of the upper atmosphere could, at least in theory, break the chemical bonds of CFCs, releasing free chlorine atoms, which react catalytically with ozone and result in its significant depletion.^7^ By then, CFCs had been in commercial production for around 40 years, and it would take 15 more years before the Montreal Protocol on Substances that Deplete the Ozone Layer, the first global treaty regulating the production and consumption of ozone-depleting substances entered into force.^8^

This long delay and the manifold amount of human-created novel entities compared to the 1930s, raises the question of how many novel entities we have nowadays in production that will, in 50 years, be considered to threaten some vital planetary boundary? The novel entities, as such without a defined boundary value is a vital reminder of this problem.^9^
